# Gait strategies for individuals with knee osteoarthritis when transitioning between floor and stair walking

**DOI:** 10.3389/fphys.2023.1026299

**Published:** 2023-01-24

**Authors:** Yatai Chai, Jian Chen, Meijin Hou, Jiaqi Zheng, Lu Liu, Yongkang Chen, Shengxing Fu, Ye Ma, Tiansheng Hong, Xiangbin Wang

**Affiliations:** ^1^ Fujian University of Traditional Chinese Medicine, Fuzhou, China; ^2^ Key Laboratory of Orthopedics and Traumatology of Traditional Chinese Medicine and Rehabilitation Ministry of Education, Fujian University of Traditional Chinese Medicine, Fuzhou, China; ^3^ Rehabilitation Hospital affiliated to Fujian University of Traditional Chinese Medicine, Fuzhou, China; ^4^ National-Local Joint Engineering Research Center of Rehabilitation Medicine Technology, Fuzhou, China; ^5^ College of Rehabilitation Medicine, Fujian University of Traditional Chinese Medicine, Fuzhou, China; ^6^ Faculty of Sports Sciences, Research Academy of Grand Health, Ningbo University, Ningbo, China; ^7^ Jinjiang city hospital, Quanzhou, China; ^8^ Rehabilitation Department of the Affiliated 3rd Peoples’ Hospital, Fujian University of Traditional Chinese Medicine, Fuzhou, China

**Keywords:** knee osteoarthritis, stair transition, motion analysis, Kinematics, fall risk

## Abstract

**Objective:** Individuals with knee osteoarthritis are at higher risk for falls during transitions between floor and stair walking due to their reduced lower extremity function. However, the adjust gait characteristics of knee osteoarthritis subjects for dealing with stair transitions have not been explored. We aimed to investigate gait strategies in individuals with knee osteoarthritis compared to age-matched individuals without knee OA during the transition between walking on floor and stairs.

**Method:** Gait of 30 individuals with knee osteoarthritis and 30 individuals without knee osteoarthritis during floor-to-stair and stair-to-floor walking transitions were accessed using a 3D motion capture system. Foot-tread clearance, and angles of lower extremity joints and trunk at Foot-tread clearance timepoint were analyzed by using biomechanical software (Visual 3D).

**Results:** Compared with asymptomatic control group, the knee osteoarthritis group demonstrated no difference in vertical Foot-tread clearance and horizontal Foot-tread clearance during stair transitions. However, ankle dorsiflexion (*p* = 0.01) decreased, hip flexion (*p* = 0.02) and trunk lateral tilt (*p* = 0.02) increased in knee osteoarthritis group during the stair ascent transition. Moreover, trunk lateral tilt to the support side (*p* = 0.003) and pelvic rotation (*p* = 0.03) increased, while hip abduction of the swing leg (*p* = 0.03) decreased during the stair descent transition in individuals with knee osteoarthritis.

**Conclusion:** Increased trunk lateral tilt and altered angle of hip may be a strategy utilized by individuals with knee OA to increase the foot clearance ability and compensate for the disease-related loss of lower extremity strength, range of motion, and balance. However, compensatory manifestations, such as the increased lateral tilt of the trunk and movement of the gravity center may enhance the risk of falls and result in more abnormal knee joint loading.

## Introduction

**FIGURE 1:**
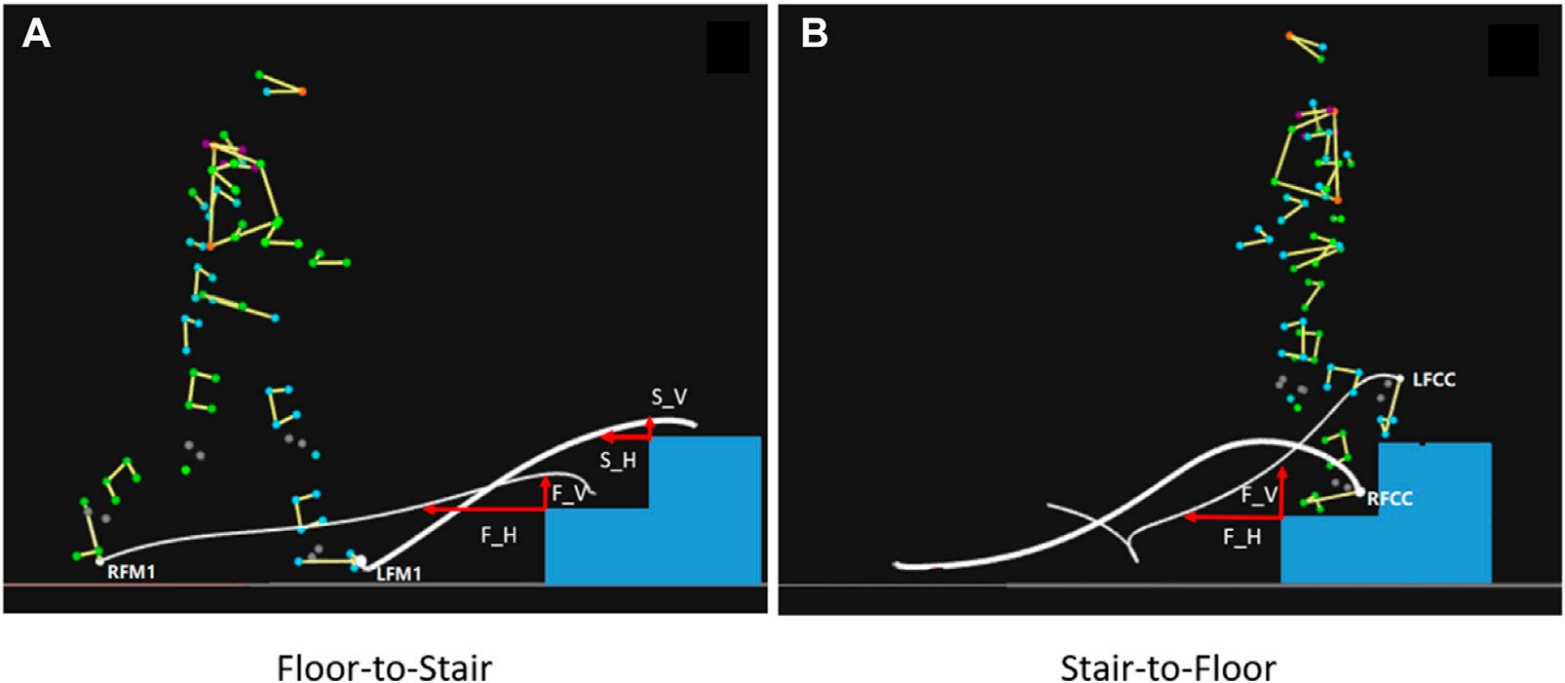
Stair transitions and Foot-tread clearance (FC). **(A)**: Floor-to-Stair transition; **(B)**: Floor-to-Stair transition. F_H: First step horizontal FC; F_V: First step vertical FC; S_H: Second step horizontal FC; S_V: Second step vertical FC. LFM1, RFM1: left and right first metatarsal head; LFCC, RFCC: left and right heels.

Stair negotiation is a more complex daily task than level walking, demands higher ranges of motion and neuromuscular control in the lower extremity (Protopapadaki et al., 2007), and increases the risk of falls (Jacobs, 2016). The transition area between floor and stairs, where occurs patterns shift from the floor to stair walking and *vice versa* (Sheehan and Gottschall, 2011), is the most common location for trips and falls (Peng et al., 2016). The transition between floor and stair walking consists of two steps, the first floor-to-stair transition step occurs from toe-off on floor to ipsilateral initial contact on the first stair, and the second step is from contralateral toe-off on the ground to initial contact on the second stair. (Alcock et al., 2014) (Figure 1A). In stair-to-floor transition, the first step is from toe-off on the second stair to ipsilateral contact with the ground. The second step is from the contralateral foot leaving the first stair to contact the ground (Figure 1B) (Alcock et al., 2015). Motor function of the lower extremities decreases with aging. The transition between floor and stair walking has also become one of the most challenging and dangerous tasks for elderly people in community life.

In senior adults, knee osteoarthritis (OA) is one of the most common degenerative diseases, causing joint pain, stiffness, and diminished stability (Lawson et al., 2015), impairing balance and increasing the risk of fall (Khalaj et al., 2014; Bozbas et al., 2017). Individuals with knee OA have reduced knee proprioception, balance responses and quadriceps strength (Mohammadi et al., 2008), which results in some restriction of daily activities. Most people with knee OA complain about difficulty when walking up and down stairs due to decreased mobility, strength, and motor control capabilities of their lower limbs. Several studies have reported that different factors, such as gender, age, and history of falls, influence exercise performance in seniors during the transition between floor and stairs (Alcock et al., 2014; Singhal et al., 2014; Di Giulio et al., 2020; Gerstle et al., 2021), but little is known about how individuals with knee OA adjust their gait in anticipation of walking-stair transitions.

Foot-tread clearance (FC) is one of critical biomechanical metrics of stair ambulation, which affects fall risk, risks of foot scuffs and tripping (Muhaidat et al., 2011). FC is defined as the minimum vertical and horizontal distances from the heel or toe of the foot to the edge of the step (Agha et al., 2021) (Figure 1). The mean minimum FC is less and the variation of FC is larger in seniors compared with those in young adults (Lythgo et al., 2007). Individuals with knee OA show a delayed quadriceps activation during stair ascent (Hinman et al., 2002). The altered kinematics during stair climbing for knee OA subjects showed larger trunk/hip flexion angles and smaller knee flexion/ankle dorsiflexion angles (Hinman et al., 2002; Iijima et al., 2018). Thus, individuals with knee OA may exhibit worse FC in the transition. The kinematic indicators that have been reported during the transition between floor and stair walking include maximum lower-limb joint angle, step width and length, etc. (Lythgo et al., 2007; Ransom et al., 2019). We investigated the angles of lower extremity joints, pelvis and trunk at the timepoint of minimum foot clearance to investigate the adjustment strategies of movement patterns in knee OA subjects associated with their gait changes in foot clearance.

The purpose of this study was to identify the altered kinematics of senior individuals with knee OA in comparison with age-matched peers during the transition phase between level-ground and stair walking. We hypothesized that individuals with knee OA would have less foot clearance and a higher risk of falls during the transition phase compared to senior adults without knee OA.

## Methods

### Subject information

We used the power of 0.8, the effect size (ES) of 0.78, and two-sided *α* = 0.05 to calculate the sample size (Lythgo et al., 2007). A minimum number of 27 participants per group is calculated *via* G × Power software (version 3.1.9.7, Franz Faul, University of Kiel). We recruited 30 subjects with knee OA and 30 asymptomatic participants (control) without knee OA, who were aged 50–70 years old, from neighboring communities. This experiment protocol was approved by the Ethics Committee of the Third People’s Hospital Affiliated with Fujian University of Traditional Chinese Medicine (FJTCM). All participants provided written informed consents before the experiment.

### Experiment protocol

**FIGURE 2:**
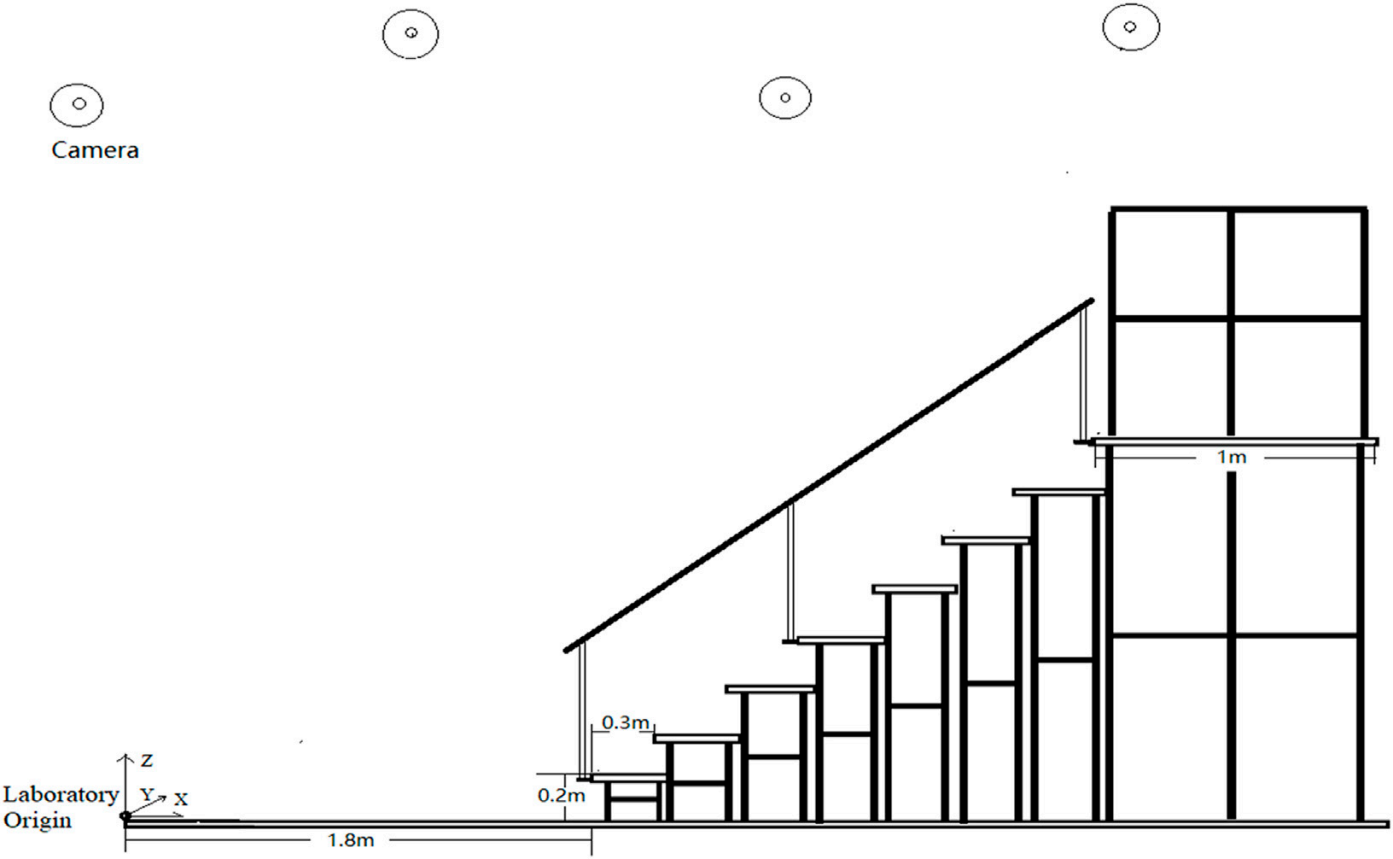
A 1.8-m walkway and an eight-step staircase. The laboratory origin is at the beginning of the walkway. The height of the stairs is 0.2 m per floor and the length is 0.3 m.

A 1.8-m walkway and a customized eight-step staircase (Liu et al., 2022) (height: 20 cm, tread depth: 30 cm, see Figure 2) with handrails on both sides were used to mimic stair transition scenarios in daily lives. At the top of the staircase, a 1-m long and 2.5-m wide platform with a fence around the platform was equipped for security.

**FIGURE 3:**
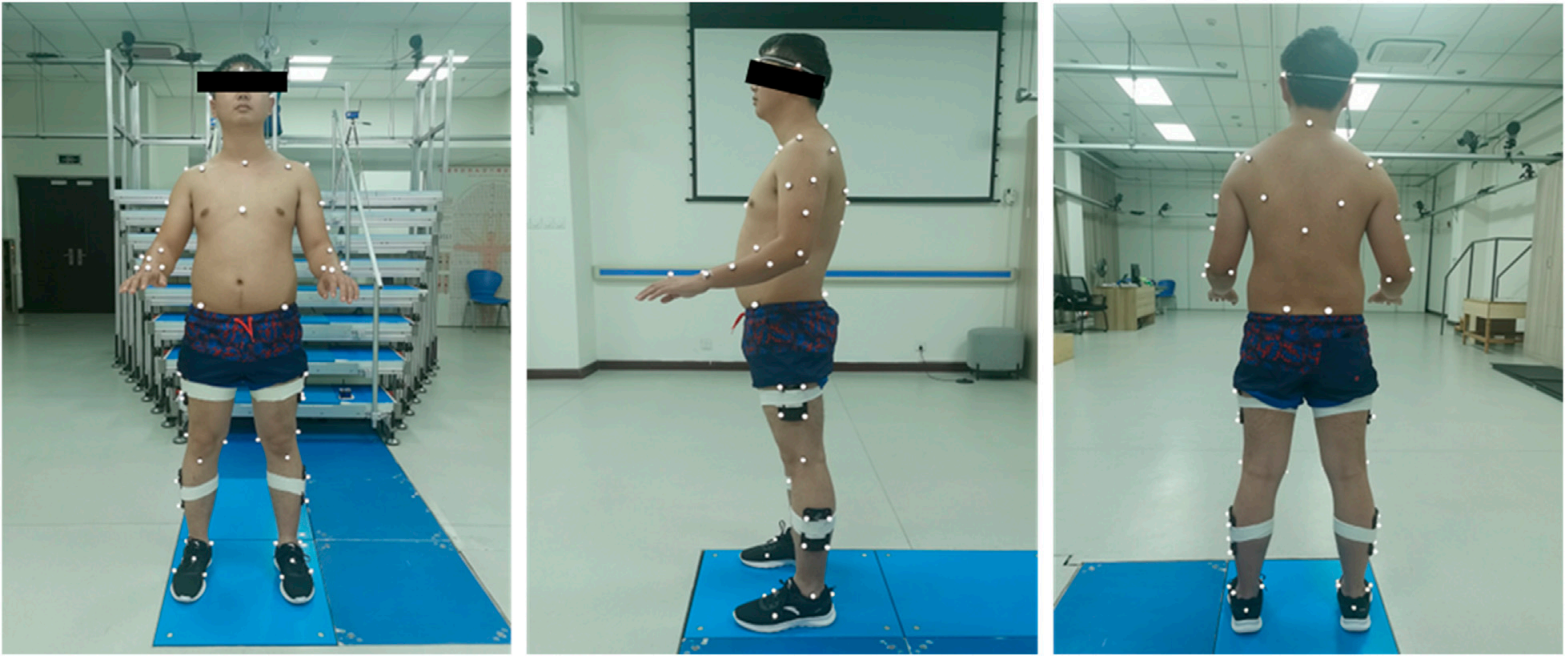
The placement of the markers.

Following the administration of the clinical tests, 75 reflective markers, according to the calibrated anatomical systems technique protocol (Cappozzo et al., 1995; Liu et al., 2022), were attached to the anatomical landmarks of each subject (Figure 3; Table.1). The four markers on the feet were secured to sports shoes. All subjects were required to wear the same shoes for standardization. A 15-segment whole-body model was developed for kinematic calculation. The marker trajectories were recorded at a sampling rate of 100 Hz by a 3D motion capture system equipped with ten infrared cameras (Oqus7+, Qualisys AB, Sweden) (Wang et al., 2020). Participants were allowed to practice the stair transitions task before testing to familiarize themselves with the environment and the task.

**TABLE 1:** Marker location.

Marker name	Marker location	Marker name	Marker location
Upper body (Legrand et al., 2021)		Lower body (38)	
L/R_HEAD	Just above the ear	L/R_IAS	Anterior superior iliac spine
SGL	Glabella	L/R_IPS	Posterior superior iliac spine
CLAV	Clavicular notch	L/R_TH1-4Cluster	Cluster of four markers placed on the lateral surface of the thigh
STRN	Sternum	L/R_FLE	Lateral epicondyle
CV7	7th Cervical Vertebrae	L/R_FME	Medial epicondyle
TV10	10th Thoracic Vertebrae	L/R_TT	Tuberositas tibiae
L/R_SIA	Scapula-Inferior Angle	L/R_SK1-4Cluster	Cluster of four markers placed on the lateral surface of the shank
L/R_SAE	Scapula-Acromial Edge	L/R_FAL	Lateral prominence of the lateral malleolus
L/R_ASH	Anterior shoulder	L/R_TAM	Medial prominence of the medial malleolus
L/R_PSH	Posterior shoulder	L/R_FCC	Aspect of the Achilles tendon insertion on the calcaneus
L/R_1-3Cluster	Cluster of three markers placed on the lateral surface of the upper arm	L/R_FM1	Dorsal margin of the first metatarsal head
L/R_HLE	Humerus– Lateral Epicondyle	L/R_FM2	Dorsal aspect of the second metatarsal head
L/R_HME	Humerus– Medial Epicondyle	L/R_FM5	Dorsal margin of the fifth metatarsal head
L/R_1-3Cluster	Cluster of three markers placed on the lateral surface of the forearm		
L/R_RSP	Radius– Styloid Process		
L/R_USP	Ulna– Styloid Process		
**L/R_HM2**	Basis of Forefinger		

Formal testing consisted of static standing trials and stair transitions trials. Static standing trials were recorded to create a model of the participant in Visual 3D. Participants were then asked to ascend the stairs to the top after passing through a 1.5-m walkway at their comfortable speeds, and then wait for the collection device to be ready at the top platform before descending back to the starting position in the formal test. The floor-to-stair transition interval begins when the toe of the lead foot leaves the floor and ends when the trailing foot touches the second stair, and the stair-to-floor transition begins when the toe of the lead foot leaves the second stair and ends when the trailing foot touches the floor (Figure 1). The test was repeated until five valid trials were collected. A valid trial is defined as passing through the transition area between stairs and floor without falling, tripping, or holding the handrail.

### Data analysis

Signal processing and kinematic calculations were performed in Visual3D (V6, C-motion Inc., Germantown, MD, United States). The marker trajectories were filtered using a zero-lag fourth-order low-pass Butterworth filter with a cutoff frequency of 6 Hz (Robertson and Dowling, 2003). Minimum foot-tread clearance was defined as the minimum distance of the foot from the edge of the step, divided by the minimum horizontal and vertical distance. During the floor-to-stair transition, the marker FM1 at the base of the first metatarsal was used as the reference point, and the event was created when the height of the marker FM1 on the Z-axis is equal to the height of the step or the distance to the laboratory origin of the FM1 on the X-axis is equal to the distance to the laboratory origin of the step edge. The distance from the step edge to the laboratory origin minus the distance from FM1 to the origin is equal to the horizontal FC, while the vertical FC is equal to the vertical height of FM1 minus the height of the step.

$$Horizontal\;FC=D_{step\;edge}-X_{FM1}$$

$$Vertical\;FC=Z_{FM1}-H_{step}$$

$X_{FM1}$ and $Z_{FM1}$ are space coordinates of the marker FM1 on the *X* and *Z*-axes. The horizontal and vertical FC during the stair-to-floor transition, with the heel marker FCC as the reference point, are calculated in the same way as above.

FC include first step horizontal FC(F_H), first step vertical FC(F_V), second step horizontal FC(S_H), and second step vertical FC(S_V) (Figure 2). Joint angles of the ankle and knee joints in the sagittal plane and angles of the hip, pelvis, and trunk joint in the sagittal, coronal, and horizontal planes at FC timepoint were derived from Visual 3D. At the FC timepoints, we not only focus on the difference in joint angle of the swinging leg across the edge of the stairs, but also the difference in the joint angle of the supporting leg at this timepoint.

### Statistical analysis

All values were presented as mean ± standard deviation (SD) or medians (interquartile ranges). The normality of the FC and angles at FC timepoint was assessed using the Shapiro-Wilk test before all analyses. Data with normal distribution were analyzed using an independent sample *t*-test, while those without normal distribution were analyzed using a Mann–Whitney U test. All statistical analyses were performed using IBM SPSS version 25.0 (SPSS, Inc., Chicago, IL, USA). Significant differences were determined by a *p*-value of less than 0.05.

## Results

### Participants

Sixty participants completed the study (knee OA group: n = 30; control group: n = 30). There was no significant difference between the groups about age (knee OA group: 58.87 ± 5.17 vs control group: 59.33 ± 5.59 years, *p* = 0.74), height (1.59 ± 0.06 vs 1.61 ± 0.07 m, *p* = 0.29), body mass (57.38 ± 6.67vs 60.01 ± 10.18 kg, *p* = 0.952), BMI (22.71 ± 2.22 vs 23.15 ± 2.67 kg/m2, *p* = 0.49; Table 2).

**TABLE 2:** Characteristics of the knee osteoarthritis (OA) and control groups.

	Knee OA (n = 30)	Control (n = 30)	*p-value*
Age	58.87 ± 5.17	59.33 ± 5.59	0.74
Height (m)	1.59 ± 0.06	1.61 ± 0.07	0.29
Body mass (kg)	57.38 ± 6.67	60.01 ± 10.18	0.24
BMI (kg/m^2^)	22.71 ± 2.22	23.15 ± 2.67	0.49

### Minimum foot-tread clearance

There was no statistical difference in horizontal and vertical FC between the knee OA and the control groups during both the floor-to-stair and stair-to-floor transitions (*p* > 0.05; Table 3).

**TABLE 3:** Minimum foot-tread clearance.

FC	Knee OA (n = 30)	Control (n = 30)	*p-value*
Floor-to-stair transition			
F _H	0.40 ± 0.07	0.41 ± 0.09	0.72
F _V	0.10 ± 0.01	0.10 ± 0.01	0.80
S _H	0.29 ± 0.03	0.28 ± 0.05	0.22
S _V	0.08 (0.08,0.09)	0.08 (0.07,0.09)	0.47
Stair-to-floor transition			
F _H	0.29 ± 0.05	0.28 ± 0.08	0.73
F _V	0.14 ± 0.02	0.13 ± 0.03	0.23

### Joint angles at FC

**FIGURE 4:**
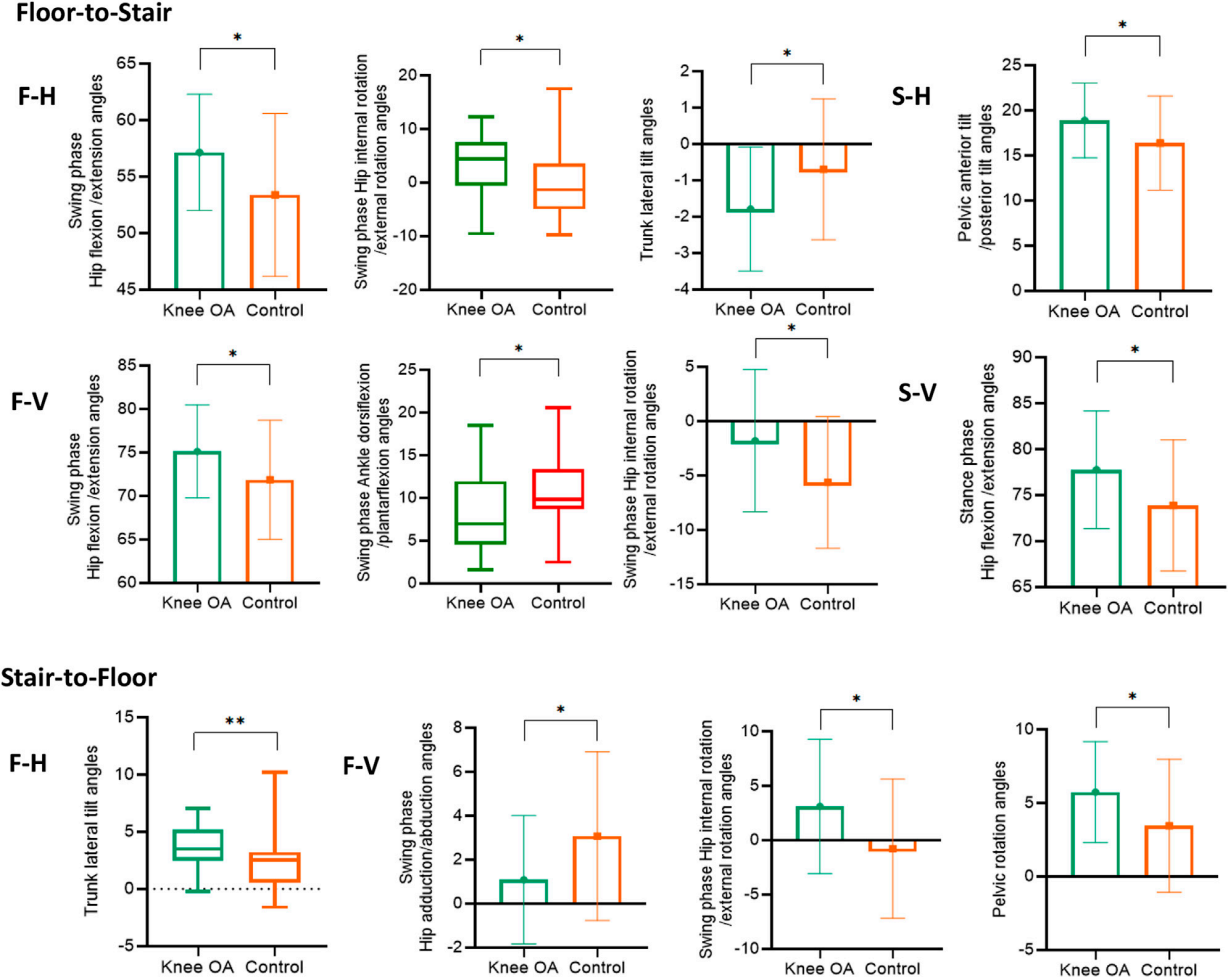
The significant differences in joint angles at FC moment in Floor-to-Stair and Stair-to-Floor transition.

During floor-to-stair transition, when the leading leg approaches the edge of first step, hip flexion (57.15 ± 5.14 vs. 53.39 ± 7.20, *p* = 0.02) and internal rotation in the swing leg (4.37 (−0.52 ,7.55) vs. −1.34 (−4.98,3.55), *p* = 0.02), and lateral tilt of the trunk (−1.79 ± 1.70 vs. −0.70 ± 1.94, *p* = 0.02) were significantly increased in the knee OA group compared with the control group. When the leading leg passes the edge of the second step, the knee OA group showed smaller ankle dorsiflexion of the swing leg (6.99 (4.55,11.97) vs. 9.84 (8.73,13.39), *p* = 0.01), along with greater hip flexion (75.12 ± 5.37 vs. 71.86 ± 6.83, *p* = 0.04) and smaller external rotation (−1.79 ± 6.54 vs. −5.62 ± 6.05, *p* = 0.02. See Figure 4) than the control group. Moreover, anterior pelvic tilt increased in the knee OA group (18.88 ± 4.15 vs. 16.37 ± 5.22, *p* = 0.04) when the trailing leg approaches the edge of second step, and hip flexion of the supporting leg increased in the knee OA group (77.76 ± 6.40 vs. 73.89 ± 7.15, *p* = 0.03. see Figure 4).

During stair-to-floor transition, smaller hip abduction in the swing leg (1.09 ± 2.92 vs. 3.07 ± 3.84, *p* = 0.03), greater external rotation (3.11 ± 6.16 vs. −0.77 ± 6.40, *p* = 0.02) and pelvic rotation (5.74 ± 3.43 vs. 3.45 ± 4.53, *p* = 0.03) were detected in the knee OA group when the leading leg passes the edge of the first step. The lateral tilt of the trunk to the side of the supporting leg obviously increased in the knee OA group at F_H timepoint (3.50 (2.48,5.18) vs. 2.54 (0.56,3.22), *p* = 0.003. see Figure 4).

## Discussion

We aimed to investigate gait strategies in individuals with knee OA during the transition phase between floor and stairs, then we found that subjects with knee OA adopted a primary strategy by increasing trunk lateral tilt and hip flexion in response to the challenge of stair-walking *versus* level-ground walking patterns shift, compensating for decreased lower extremity function, resulting in no difference in FC.

Transitions between floor and stair walking are an anticipation of the next stride (Sheehan and Gottschall, 2011), the body adjusts strategies to respond to task changes, such as increasing foot clearance (Prentice et al., 2004). At the beginning of the floor-to-stair transition, the swing leg which passes the edge of the first step requires greater hip flexion, knee flexion, and ankle dorsiflexion to allow the toes to pass safely over the step edge compared with the level-ground walking (Madsen and Frandsen, 1989). The foot clearance ability can be used as an indicator of the lower limb motor control ability during stair walking. Individuals with knee OA had reduced lower extremity joint mobility and decreased strength of the knee extensors, hip external rotation and abduction muscles, and ankle internal rotation muscles compared to healthy individuals of the similar age (Øiestad et al., 2015; Park et al., 2016). That may cause reduced FC in individuals with knee OA. Levinger et al. reported that FC has not been shown different between individuals with knee OA and healthy older people, but the knee OA group used a different strategy to achieve the same foot clearance in floor walking, manifested as greater knee flexion, greater hip abduction and less ankle adduction (Levinger et al., 2012). This is similar to our results. Although gait strategies are used to achieve the same foot clearance as normal people, knee OA individuals seems to adopt the strategy more achieved through the hip joint in the transitions between floor and stair walking.

Hip flexion, hip internal rotation and trunk lateral tilt increased in individual with knee OA at F_H timepoint in the mid-swing phase, this was effective in increasing FC (Ribeiro et al., 2019). Ankle dorsiflexion decreased in subjects of knee OA group at F_V timepoint in the late swing phase, it may associate with a decreased activation level of tibialis anterior muscle (Fu et al., 2021), increasing hip flexion and decreasing hip external rotation could compensate for the decreased FC which caused by the decrease in ankle dorsiflexion. The anterior pelvic tilt angle increased when the trailing leg approached the second step edge in individuals with knee OA. Previous study shows decreased abdominal muscle strength in individuals with knee OA (D Maryama, 2018). Another study has shown that transversus abdominis activation does not alter gait impairments in people with and without knee OA (Flowers et al., 2021), but this study only investigated on floor gait. The abdominal muscles may need to be activated more for control trunk balance and lumbar stabilization when ascending stairs, reducing excessive lumbar lordosis or pelvic anterior tilt will help improve the function of the musculoskeletal system and movement stability (Lee, 2019). The increased hip flexion of the supporting leg may also provide an upward momentum for the stair-climbing task when the swing leg passes the edge of the second step.

Greater eccentric contraction of the knee extensors is required during the transition from stair descent to floor walking. Individuals with knee OA increased pelvic posterior tilt at the timepoint of F_V, with reduced hip abduction and increased external rotation, which may be related to weaker hip abductor strength (Costa et al., 2010; Hinman et al., 2010). The F_H timepoint is event at the late swing phase, and the knee OA subjects increased lateral tilt of the trunk toward the side of the supporting leg. Damage to the lumbar region and movement of center of gravity may be caused by the increased trunk lateral tilt, which significantly increases the frontal moment at the hip (Legrand et al., 2021), leads to abnormal load distribution in the internal and external compartments of the knee joint and accelerate the progression of knee OA.

A limitation must be acknowledged. Kinematic changes we found in stair walking transitions may be closely related to muscle activity, but we had not collected surface electromyography data. In the future, we will continue to explore the muscle activity during stair walking transitions in individuals with knee OA.

## Conclusion

We found that individuals with knee OA have a different motor control pattern compared with age-matched asymptomatic peers during the transitions between floor and stair walking, with the increased lateral tilt of the trunk and hip flexion to keep almost normal foot clearance. During the stair-to-floor transition, increased lateral tilt of the trunk to the supporting side is adopted to compensate for the lack of gluteus medius strength. However, the risk of falls is also increased by these compensatory manifestations as increased lateral tilt of the trunk, or larger movement of gravity center, raising the possibility of other impairment such as more abnormal knee joint loading A subject-specific rehabilitation program like strengthening gluteus medius strength may help improve motor control for individuals with knee OA during stair transfers and reduce their incidence of falls.

## Data Availability

The original contributions presented in the study are included in the article/supplementary material, further inquiries can be directed to the corresponding author.
